# Heterologous Gene Expression System Using the Cold-Inducible *CnAFP* Promoter in *Chlamydomonas reinhardtii*

**DOI:** 10.4014/jmb.2007.07024

**Published:** 2020-08-15

**Authors:** Minjae Kim, Jongrae Kim, Sanghee Kim, EonSeon Jin

**Affiliations:** 1 Department of Life Science, Research Institute for Natural Sciences, Hanyang University, Seoul 04763, Republic of Korea; 2 Division of Polar Life Science, Korea Polar Research Institute, Korea Institute of Ocean Science and Technology, Incheon 1990, Republic of Korea

**Keywords:** Cold-inducible expression system, antifreeze protein, truncated promoter, *Chlamydomonas reinhardtii*, *Gaussia luciferase*, fluorescent protein

## Abstract

To increase the availability of microalgae as producers of valuable compounds, it is necessary to develop novel systems for gene expression regulation. Among the diverse expression systems available in microalgae, none are designed to induce expression by low temperature. In this study, we explored a cold-inducible system using the antifreeze protein (AFP) promoter from a polar diatom, *Chaetoceros neogracile*. A vector containing the *CnAFP* promoter (*pCnAFP*) was generated to regulate nuclear gene expression, and reporter genes (*Gaussia luciferase* (*GLuc*) and *mVenus* fluorescent protein (*mVenus*)) were successfully expressed in the model microalga, *Chlamydomonas reinhardtii*. In particular, under the control of *pCnAFP*, the expression of these genes was increased at low temperature, unlike *pAR1*, a promoter that is widely used for gene expression in *C. reinhardtii*. Promoter truncation assays showed that cold inducibility was still present even when *pCnAFP* was shortened to 600 bp, indicating the presence of a low-temperature response element between –600 and –477 bp. Our results show the availability of new heterologous gene expression systems with cold-inducible promoters and the possibility to find novel low-temperature response factors in microalgae. Through further improvement, this cold-inducible promoter could be used to develop more efficient expression tools.

## Introduction

Microalgae are potential sustainable and natural feedstocks for future industries, with applications in the production of biodiesel, pharmaceuticals, and cosmetics [1-3]. Despite numerous attempts to optimize culture conditions and increase the productivity of valuable compounds [4, 5], the use of wild-type strains places limits on the variety of compounds that can be produced, and the productivity of these strains is also bounded [6]. Therefore, the improvement of production strains is required, and the development of novel methods to regulate gene expression has become an important consideration [1, 7].

Gene promoter regions are important for the initiation of gene expression. Several types of promoters have been developed to express heterologous genes in microalgae [8]. In *Chlamydomonas reinhardtii*, a model green microalga, promoter systems that regulate gene expression are particularly well developed and include the promoters of *β-2-tubulin* [9], *HSP70A* [10], *RbcS2* [11], *AR1* [10], and *Lhcb-1* [12]. The *AR1* promoter, which is a chimera of the *HSP70A* and *RbcS2* promoters, has been used widely due to its high constitutive gene expression [10, 13]. However, the *AR1* promoter cannot be used in expression systems where gene expression levels need to be modulated according to specific conditions. Unlike constitutive promoters, inducible promoters regulate gene expression when cells are exposed to specific conditions. In *C. reinhardtii*, the high-temperature–inducible promoter (*HSP70A*) and light-inducible promoter (*LIP*) are activated under heat and high light stress conditions, respectively [14, 15]. The *HSP70A* promoter is activated at 40°C, and the *LIP* promoter is activated by exposure to more than 500 μmol photons m^–2^ s^–1^.

Generally, high temperature caused by heat or high light stresses can affect the stability of protein structures and protein solubility. In the case of bacterial systems, cold-inducible promoters such as the cold-shock protein A (*cspA*) promoter are used to overexpress proteins at low temperature, thereby increasing protein solubility and stability [16]. However, cold-inducible promoters that can be used in *C. reinhardtii* have not yet been developed [17]. Recently, there have been attempts to produce useful recombinant proteins such as human growth factor, interferon β, or proinsulin using microalgae [18-20], so the development of cold-inducible expression systems may be necessary. Given its successful application in bacterial systems, the development of a vector system that uses a cold-inducible promoter might increase the efficiency of protein production in microalgae.

Antifreeze protein (AFP), which acts as antifreeze in some organisms, is an important defense mechanism against cold stress in organisms living in the polar regions [21, 22]. In our own previous research, the *AFP* sequence of polar diatom *Chaetoceros neogracile* was found using an expressed sequence tag database [23, 24]. In addition, its promoter sequence was obtained in the 5’ upstream region of the *AFP* gene through inverse PCR [24, 25]. Interestingly, the protein expression of *AFP* was enhanced in response to temperature and light stresses [24-26]. Therefore, the promoter of *CnAFP* is thought to be useful for controlling heterologous gene expression in microalgae through temperature downshifts.

In this study, we aimed to develop a cold-inducible vector system using the *AFP* promoter of *C. neogracile* (*pCnAFP*) in *C. reinhardtii*. First, we obtained the promoter sequence from the genomic DNA of *C. neogracile* and constructed vectors containing either the *Gaussia luciferase* gene (*GLuc*) or the *mVenus* fluorescent protein gene (*mVenus*). Each reporter system was introduced into *C. reinhardtii* to confirm the regulation of expression by *pCnAFP*. Low-temperature responsiveness of these vectors was confirmed from the quantitative real-time PCR of *GLuc* and *mVenus*, and the luciferase assay of *GLuc*. Furthermore, we confirmed the minimum length of the cold-inducible promoter through a progressive truncation assay, and this work therefore supports the potential application of cold-inducible gene expression in *C. reinhardtii*.

## Materials and Methods

### Algal Strain and Standard Culture Conditions

The green microalga *Chlamydomonas reinhardtii* CC–4349 (cw15 mt^–^) was cultured in Tris-acetate-phosphate (TAP [27]) medium with shaking on an orbital shaker at 90 rpm under continuous white fluorescent light (75 ± 10 μmol photons/m^–2^ s^–1^) at 25°C. To select transformants, cells were grown on solid TAP medium plates containing 1.5% agar with hygromycin B (25 μg/ml).

### Isolation of the *CnAFP* Promoter and Prediction of Transcriptional Regulatory Elements

Based on the promoter region data on the *CnAFP* gene described in our previous paper [25], we obtained the promoter sequence through PCR from the genomic DNA of *C. neogracile* using a gene-specific primer set. The promoter fragment was cloned into the *pBlunt*-TOPO vector (MG Blunt TOPO Cloning Kit, Cancer Rop Co., Korea). The sequence of the cloned promoter region was verified by Sanger sequencing (Macrogen, Korea). The primers used for the isolation of the 1,225 bp *CnAFP* promoter and sequencing are listed in Table S1. In order to predict the transcriptional regulatory elements, the promoter sequence was investigated by using the following analysis tools: PLACE [28], PlantPAN [29], PlantCARE [30], and Softberry [31].

### Vector Construction

We used the previously reported *pChlamy3*_*GLuc* as the vector backbone [32]. It was based on the *pChlamy3* vector from the GeneArt Chlamydomonas Engineering Kit (Life Technologies, USA) and was designed to express the codon-optimized *Gaussia luciferase* (*GLuc*) gene according to the linked promoter sequence. As a selective marker of the *pChlamy3* vector, a hygromycin resistance gene provides stable resistance against hygromycin B, a commonly used antibiotic [33]. Also, the *GLuc* was selected as a reporter protein to confirm the functionality of the constructed vector in *C. reinhardtii* [34]. The PCR products of the *CnAFP* promoter were inserted between the *SpeI* and *KpnI* sites of the MCS2 site. To express the fluorescent protein *mVenus*, the *CnAFP* promoter was cloned into the *pChlamy3*_*mVenus* vector by fragment replacement. The primers used to generate expression vector constructs are listed in Table S1.

### Generation of Transgenic Chlamydomonas

Expression vectors were introduced into *C. reinhardtii* by electroporation following the protocol provided with the GeneArt Chlamydomonas Engineering Kit with slight modifications. The cells were grown to early exponential phase in TAP medium and 1 ml of culture (5 × 10^6^ cells) was harvested by centrifugation at 2,000 ×*g* for 2 min. The cell pellet was resuspended in 0.25 ml of TAP medium containing 40 mM sucrose, to which vector DNA linearized by SpeI was added. The mixture was transferred into an electroporation cuvette (4 mm) and incubated for 5 min at room temperature. Electroporation was conducted with the selected parameters (600 V, 50 μF, and 200 Ω) using the Bio-Rad Gene Pulser X cell apparatus (Bio-Rad, USA). Immediately after electroporation, the cuvette was cooled on ice for 5 min. Then, the cells were transferred to 10 ml round-bottom tubes with up to 2 ml of TAP medium and incubated for more than 12 h at 25°C in the dark. After incubation, the cells were mixed with 2 ml of melted 0.5% agar cooled to 45°C, and then spread on solid TAP plates containing 25 μg/ml hygromycin. Putative transgenic cells formed colonies after 7–10 days and were tooth-picked to small volumes of liquid TAP medium. Insertion of transformed genes was confirmed using colony PCR according to a previous paper [15].

### RNA Extraction and Quantitative Real-Time PCR

Transformants grown to mid-exponential phase under standard culture conditions were incubated in the dark for 6 h to remove the effects of other stress stimuli. Then, they were transferred to dark and low-temperature conditions (20°C, 10°C, or 0°C). After 2 h, 5 ml of low-temperature-treated cells was harvested and frozen to extract total RNA using the RNeasy Plant Mini Kit (Qiagen, Germany). cDNA synthesis was performed using reverse transcription with 2× reverse transcription master premix (ELPiS Biotech, Korea). Synthesized cDNA was amplified using SYBR premix (Takara, Japan) and the Thermal Cycler Dice Real Time System TP 8200 (Takara). The mRNA levels of *GLuc* and *mVenus* were compared between samples and normalized to that of the *receptor of activated protein C kinase 1* (*RACK1*) gene. Results were analyzed by the ΔΔCt method. Sequences of primers used for quantitative real-time PCR are listed in Table S1.

### Luciferase Activity Assay

Each transgenic *C. reinhardtii* sample in the mid-exponential phase was prepared with the same cell density based on absorbance (OD_750nm_ = 1.0). The cells were incubated in the dark for 12 h to remove the effects of other stress stimuli, and then transferred to dark and low-temperature conditions (20°C, 10°C, or 0°C). After low-temperature treatment, cells were harvested by centrifugation at 13,000 ×*g* for 2 min. Luciferase assays were performed using a Renilla Luciferase Assay Kit (Promega, USA) with a modified protocol [15]. Cell pellets were resuspended in 100 μl of 1× lysis buffer and vortexed vigorously for 3 min. After centrifugation at 13,000 ×*g* for 5 min (4°C), 90 μl of supernatant and 10 μl of 1× luciferase substrate were mixed in a new 1.5 ml tube. Immediately after mixing, the luminescence was measured using a GloMax 20/20 (Promega).

### Fluorescence Microscopy Analysis

Representative transformants were cultivated in TAP medium and exposed to low temperature (10°C) as above. Live cells were dropped on a glass slide and covered with a coverslip. Fluorescence of *mVenus* was detected using green fluorescence under a Nikon Eclipse Ni fluorescence microscope (Nikon, Japan). Fluorescence detection wavelengths were 540 ± 20 nm with the YFP filter for *mVenus* and 630 ± 30 nm with the Texas RED filter for chloroplast auto-fluorescence.

## Results and Discussion

### Response of the *CnAFP* Promoter Under Low-Temperature Conditions

**Fig. 1:**
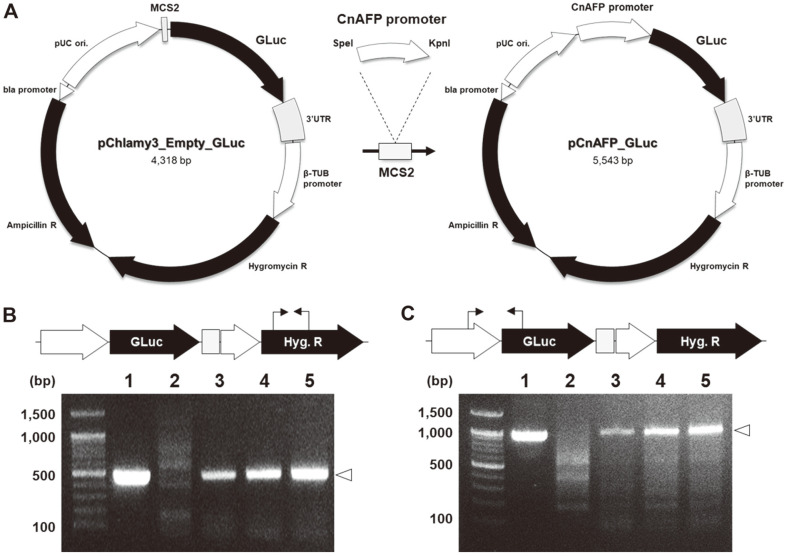
Generation of the *pCnAFP* vector construct and verification of transformants. (**A**) Vector backbone *pChlamy3*_*Empty*_*GLuc* (left) does not have a promoter sequence in front of the *Gaussia luciferase* gene (*GLuc*). Vector backbone *pCnAFP*_*GLuc* (right) with the 1,225 bp *pCnAFP* insertion. (**B** and **C**) Confirmation of vector insertion in transformants by colony PCR (**B**, hygromycin resistance gene, 467 bp; **C**, region connecting *pCnAFP* and *GLuc*, 964 bp). Lanes 1: plasmid positive for *pCnAFP*_*GLuc*, lanes 2: wild-type *C. reinhardtii*, lanes 3–5: *pCnAFP*_*GLuc* transformants.

With the *pCnAFP*_*GLuc* vector (Fig. 1A), we transformed *C. reinhardtii* and isolated the putative transformants that formed colonies on a solid agar plate containing hygromycin B (25 μg/ml). We then used the collected colonies to perform PCR with diverse primer sets to verify vector insertion and confirm the presence of bands of expected sizes (Figs. 1B and 1C). This analysis confirmed that we had obtained the putative transformants with the *pCnAFP*_*GLuc* vector inserted into the genomic DNA of *C. reinhardtii*.

**Fig. 2:**
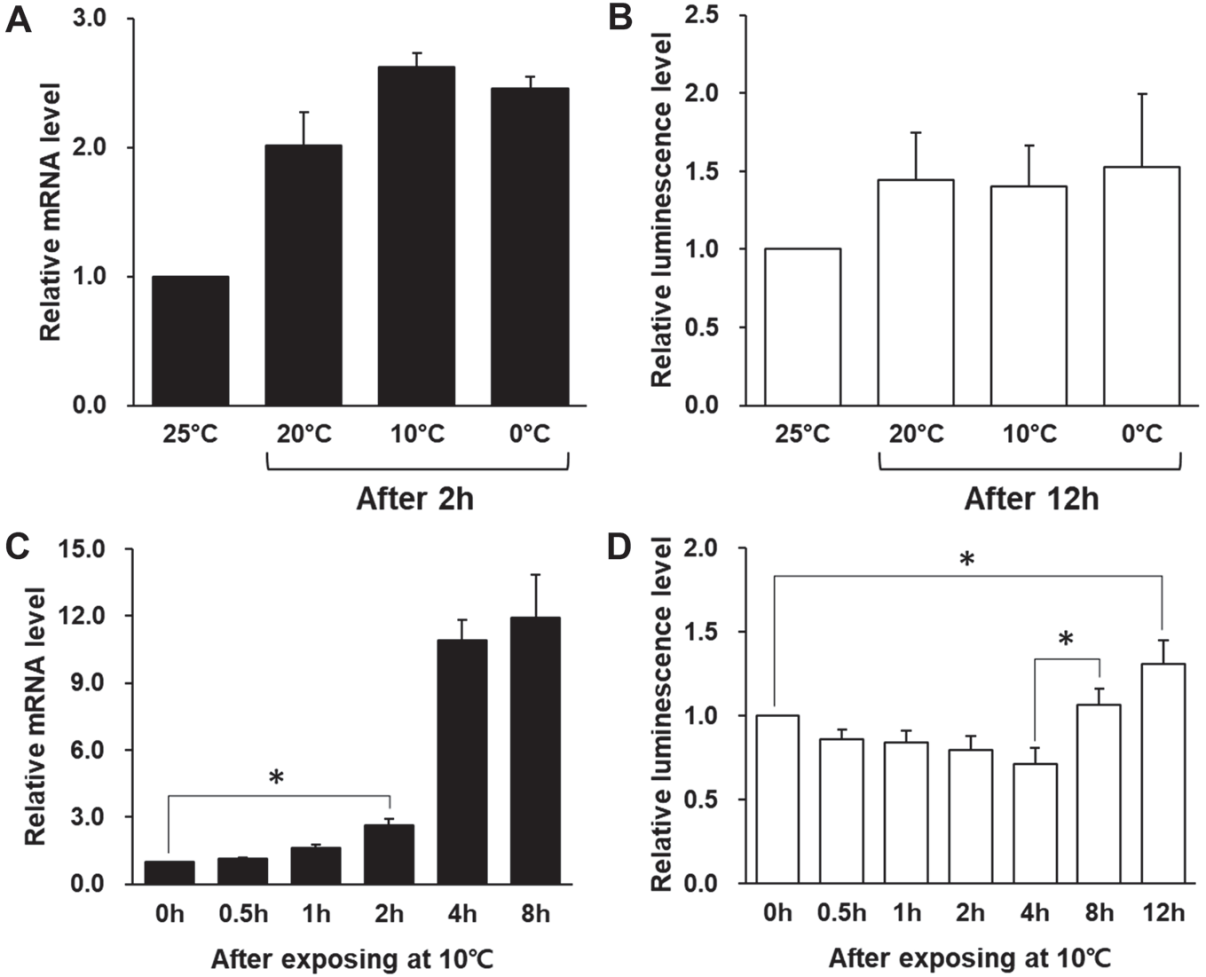
Inducibility test of *pCnAFP*_*GLuc* transformants. (**A**) Relative *GLuc* mRNA levels after 2 h treatment at the low-temperature conditions; (**B**) Relative luminescence levels after 12 h treatment at the low-temperature conditions; (**C**) Relative *GLuc* mRNA levels at 10°C; (**D**) Relative luminescence levels at 10°C. (**A**) and (**B**) were presented with the relative value calculated by the value at the optimal culture temperature of *C. reinhardtii* (25°C). All experiments were conducted with at least four replicates. Statistical analyses were performed using the Student’s *t*-test, *p < 0.05.

To determine the appropriate experimental methods and conditions to detect cold inducibility in transgenic *C. reinhardtii*, we conducted transcriptional analysis and enzyme activity assays. First, we observed the levels of *GLuc* expression and luciferase activity at several temperature conditions to determine the cold-inducible temperature of the *pCnAFP* vector. Exposure to 10°C induced the highest level of *GLuc* gene expression, with a 2.6-fold increase in comparison with 25°C (Fig. 2A). This pattern was similar to the result of the luminescence signal that indicates a readout for *GLuc* enzyme activity, in which the relative luminescence level increased about 1.5-fold relative to 25°C when the transformants were exposed to 10°C (Fig. 2B). On the basis of these results, we chose 10°C as the temperature for confirming the cold inducibility of the *CnAFP* promoter in further experiments.

Next, we examined time-dependent changes in the levels of *GLuc* mRNA expression and luminescence to choose the optimal sampling time point for assessing the cold inducibility of *pCnAFP*. The mRNA level gradually increased after a temperature shift to 10°C and was more than 2.6-fold the control level at 2 h (Fig. 2C). This transcriptional pattern controlled by *pCnAFP* in *C. reinhardtii* was similar to the results of previous studies [24, 25], suggesting the cold inducibility of *pCnAFP* in *C. neogracile*. The relative mRNA level of *GLuc* increased sharply at the 4 h and 8 h time points (Fig. 2C), but this increase of relative value was influenced by the expression level of reference gene (*RACK1*) decreasing 70% from the initial time point (Fig. S1). Therefore, it was confirmed that the 2 h time point is suitable for comparing cold inducibility in the mRNA expression of *GLuc*.

Meanwhile, the luminescence signal did not show cold inducibility in a short time (Fig. 2D). The luminescence signal gradually decreased until 4 h and then increased at 8 h and 12 h. Compared to 4 h and 12 h, the luminescence signal appears to increase 1.8-fold, but only 1.3-fold compared to 0 h and 12 h. We investigated the *GLuc* activity for up to 24 h after exposure at 10°C, but the luminescence level did not increase after 12 h (data not shown). As shown in Fig. 2, the increase of luminescence level occurred 6 h after the transcription level induction. This might be related to the time spent from transcription to translation. In addition, it is reported that short-term and long-term cold stresses affect *C. reinhardtii* in diverse aspects [35, 36]; particularly, they cause cell growth inhibition, membrane damage, and downregulation of some ribosome-related pathway. Therefore, cold stress may also affect protein synthesis, degradation, and secretion of *GLuc* in *C. reinhardtii* [37]. Consequently, the luciferase assay was not suitable for clear confirmation of cold inducibility in a short time, and therefore it was excluded from the following cold-inducibility test with truncated promoters.

### Comparison of the *CnAFP* Promoter with the *AR1* Promoter

**Fig. 3:**
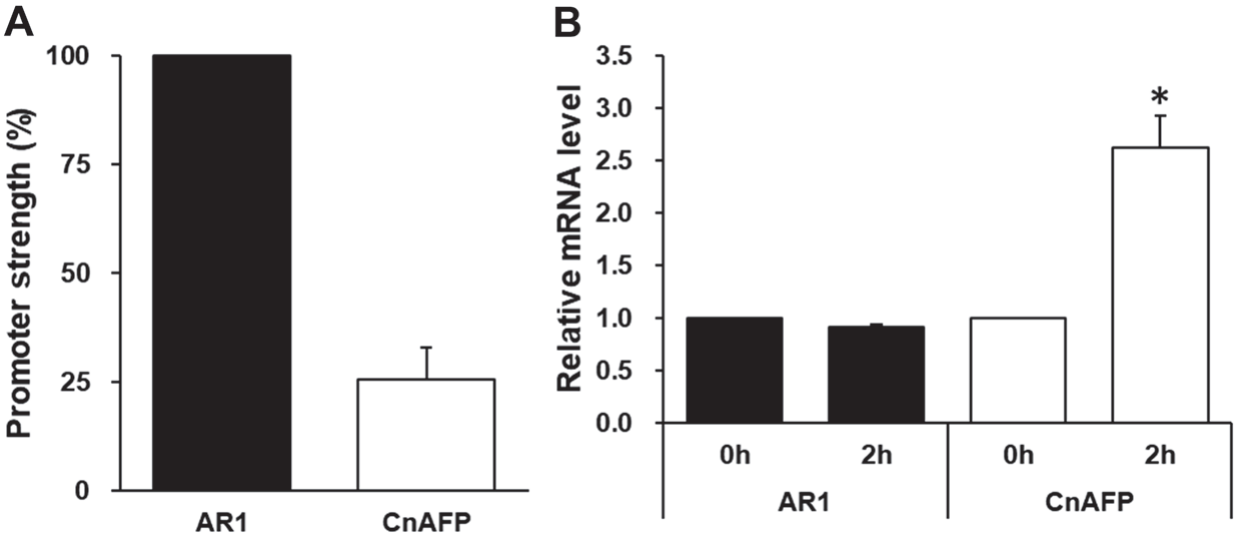
Comparison of the strength and cold inducibility of *pCnAFP* and *pAR1*. (**A**) Promoter strength of *pCnAFP* and *pAR1* based on the relative mRNA expression of *GLuc* at 25°C. The expression level of *pCnAFP* was calculated to relative value based on that of pAR1; (**B**) Relative levels of *GLuc* mRNA expression driven by *pCnAFP* and *pAR1* at 10°C. In (**B**), cold inducibility was calculated by comparing to each value in 25°C of *pAR1* and *pCnAFP*. All experiments were conducted in more than triplicate. Statistical analyses were performed using the Student’s *t*-test, *p < 0.05.

We assessed the strength of *pCnAFP* by comparing it to the *AR1* promoter (*pAR1*), a well-known constitutive promoter for *C. reinhardtii* transformation. The sequences of both vectors were identical except for the promoter regions. *GLuc* expression driven by *pCnAFP* was 25% of the level of *pAR1* (Fig. 3A). Like other heterologous promoters, pCnAFP has lower gene expression than pAR1, a strong endogenous promoter of *C. reinhardtii* [38]. However, *GLuc* mRNA expression in the *pCnAFP*_*GLuc* transformants increased 2.6-fold at 10°C compared to 25°C, unlike that in pAR1_*GLuc*, which did not change (Fig. 3B). These results indicate that *pCnAFP* has low strength, but it can be used in a cold-inducible system unlike pAR1.

### Progressive Truncation Assay of the *CnAFP* Promoter

**Fig. 4:**
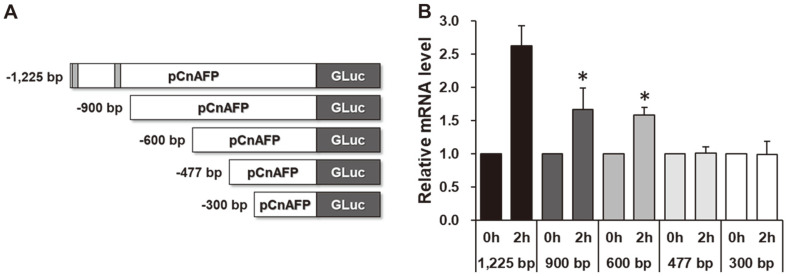
Cold-inducibility test of transformants carrying progressively truncated *pCnAFP*_*GLuc* variants. (**A**) Schematic representation of truncated promoters; (**B**) Relative levels of *GLuc* expression driven by truncated *pCnAFP*s in response to exposure to 10°C. Cold inducibility was confirmed by calculations based on the value of 25°C. All experiments were conducted with at least four replicates. Statistical analyses were performed using the Student’s *t*-test, *p < 0.05.

To apply an expression vector system more effectively, vector optimization is necessary. Considering that *pAR1* has high and constitutive expression levels in *C. reinhardtii* after being optimized [10], it might be possible to improve *pCnAFP* through sequence optimization. However, two low-temperature response element (LTRE) motifs (CCGAAA) that have been reported in the COR15a [39] and BN115 promoters [40, 41] of higher plants were predicted at the end of the 5′ region of *pCnAFP* (Table S2). Therefore, it was necessary to confirm cold inducibility in the presence of the LTRE regions. A 900 bp *pCnAFP* segment, which contained no LTRE regions, was cloned and investigated for low-temperature responsiveness. The level of *GLuc* expression driven by the 900 bp *pCnAFP* responded to low temperature similarly to that of the 1,225 bp *pCnAFP* segment (Fig. 4), although the fold increase of 900 bp *pCnAFP* was reduced from that of 1,225 bp *pCnAFP*. This result indicates that the cold inducibility of *pCnAFP* might be influenced by other transcriptional regulatory elements in addition to the LTRE motifs.

Next, we further truncated *pCnAFP* to determine the minimum length of the cold-inducible promoter. First, we made two variants of *GLuc* expression vectors; a 600 bp *pCnAFP* variant and a 300 bp *pCnAFP* variant. The 600 bp *pCnAFP* variant showed a significant increase in *GLuc* expression levels following low-temperature treatment, whereas the levels of transcription driven by the 300 bp variant remained unchanged at 10°C (Fig. 4). Since the gene expression driven by the 300 bp *pCnAFP* did not increase at 10°C, we expected that the unknown transcriptional regulatory elements related to cold/freezing conditions were located between –300 and –600 bp. However, low-temperature response elements were not predicted between –300 and –600 bp in the PLACE, PlantPAN, and PlantCARE databases (Table S2). To identify the unknown transcriptional regulatory elements in this region, the *pCnAFP* sequence was analyzed further with the TSSP (Recognition of human Pol II promoter region and start of transcription) tool using the RegSite Plant database on the Softberry web server [31]. Two regions similar to soybean embryo factor-4 binding sites (SEF4-BS: RTTTTR) were found at –497 bp and –547 bp of *pCnAFP*. In higher plants, SEF4-BS is a consensus sequence found in the promoter region of beta-conglycinin, which is involved in chilling stress response during soybean germination [42]. Thus, this region was expected to have transcriptional regulatory elements for cold inducibility.

Based on the predicted result, we made a *GLuc* expression variant that truncated the SEF4-BS-like region (477 bp *pCnAFP*), and the RNA expression in the transformants of 477 bp *pCnAFP* did not increase at 10°C (Fig. 4). This result showed that the SEF4-BS-like region could be involved in the cold inducibility of *pCnAFP*. However, the function of the SEF4-BS-like region and the factors interacting with it in microalgae are unknown, and further investigation is needed to verify that this region is the exact cold-inducible transcriptional regulatory element in microalgae. Considering that gene expression in response to low temperature is regulated by the interaction of diverse transcriptional regulatory elements in higher plants [43], it is necessary to confirm which elements interact with SEF4-BS-like regions.

Introducing longer vectors tends to be more difficult in comparison with shorter vectors, and 600 bp *pCnAFP* might be an effective cold-inducible promoter. Therefore, 600 bp *pCnAFP* was used for further comparative experiments as an optimized vector.

### Expression of Fluorescent Protein Using the *CnAFP* Promoter

**Fig. 5:**
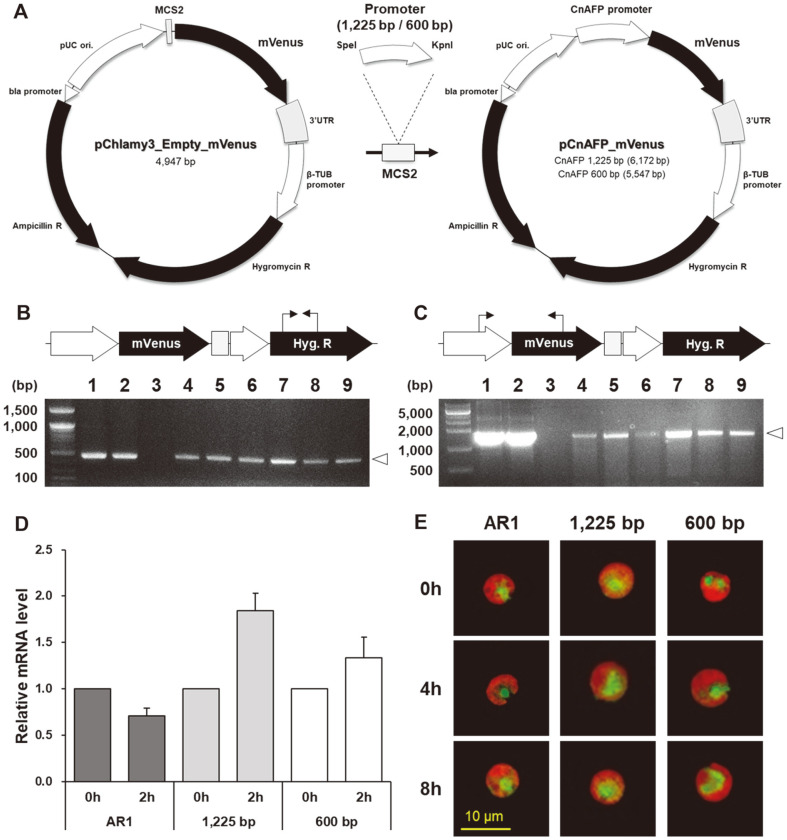
Expression of *pCnAFP*_*mVenus* in *C. reinhardtii* under low-temperature conditions. (**A**) Vector backbone of *pChlamy3*_*Empty*_*mVenus* (left) does not have a promoter sequence in front of the *mVenus* fluorescent protein gene (*mVenus*). Vector backbone of *pCnAFP*_*mVenus* (right) with either 1,225 bp *pCnAFP* or 600 bp *pCnAFP*. (B and C) Confirmation of vector insertion in transformants by colony PCR with two primer sets (B, 467 bp for hygromycin resistance gene; C, 1,519 bp for the region connecting *pCnAFP* and mVenus). Lanes 1: plasmid positive for *pCnAFP* 1,225 *bp*_*mVenus*, lanes 2: plasmid positive for *pCnAFP* 600 *bp*_*mVenus*, lanes 3: wild-type *C. reinhardtii*, lanes 4–6: *pCnAFP* 1,225 *bp*_*mVenus* transformants, lanes 7–9: *pCnAFP* 600 *bp*_*mVenus* transformants. (**D**) Changes in the relative levels of *mVenus* mRNA in response to low temperature (10°C). Cold inducibility was confirmed by calculating mRNA levels, which are relative to those at 25°C. All experiments were conducted in more than triplicate. (**E**) Fluorescent images of transformants after low-temperature treatment (0h, 4h, and 8h). Merged images of *mVenus* (emission at 537–559 nm, excitation at 502–522 nm) and chlorophyll fluorescence (emission at 603–648 nm, excitation at 563–588 nm) are shown.

To verify the expression of diverse heterologous genes other than *GLuc*, we made another vector encoding *mVenus*, which is a widely used fluorescent protein, as a reporter protein in *C. reinhardtii* [44, 45]. We made two *pCnAFP* vectors (1,225 bp and 600 bp) and a *pAR1* vector (positive control) through conjugation of the promoter–mVenus cassette to the *pChlamy3* backbone (Fig. 5A). As with *GLuc*, we confirmed vector insertion in transformants selected from solid agar plates through colony PCR (Figs. 5B and 5C).

The low-temperature responsiveness of the vectors was investigated by examining changes in mRNA levels and by fluorescence microscopy analysis. To determine mRNA levels, cells were collected at 0 h and 2 h after shifting from 25°C to 10°C. The patterns of changes in the relative expression levels of *mVenus* were clearly distinct between *pCnAFP*s and *pAR1* (Fig. 5D). Similar with the *GLuc* results, the *pCnAFP* transformants showed an increase in gene expression over time, whereas the *pAR1* transformants showed a decrease. However, the protein expression was not consistent with mRNA expression patterns. Although the fluorescent signal of the *mVenus* protein was detected in some cells of transformants, we could not confirm an increase of fluorescence according to the passage of time (Figs. 5E and S2). As in the case of *GLuc*, the *mVenus* protein synthesis in *C. reinhardtii* may have been affected by downregulation of some ribosome-related pathway at low-temperature conditions [36]. Moreover, protein synthesis regulated by *pCnAFP* can be affected by various other factors, such as mRNA stability at low temperature [46] and low promoter strength of *pCnAFP*s (Fig. 3A). Therefore, this cold-inducible promoter needs to be improved in terms of efficiency in consideration of these factors.

## Conclusion

In this work, we made a new cold-inducible expression vector using the promoter sequence obtained from the polar diatom *Chaetoceros neogracile*. The cold inducibility of this *CnAFP* promoter was confirmed by analysis of the mRNA expression of reporter genes (GLuc and mVenus) in *Chlamydomonas reinhardtii*. Consequently, our study demonstrates the availability of a cold-inducible heterologous promoter in *C. reinhardtii*, and also suggests a promising new transcriptional regulatory element responsive to low temperature. However, the strength and sensitivity of *CnAFP* promoter are not sufficient to regulate the protein expression in *C. reinhardtii*, therefore, further improvement is necessary through fusion with a stronger promoter or insertion of multiple key transcriptional regulatory elements.

## Supplemental Materials



Supplementary data for this paper are available on-line only at http://jmb.or.kr.

## References

[1] Potvin G, Zhang Z (2010). Strategies for high-level recombinant protein expression in transgenic microalgae: a review. Biotechnol. Adv..

[2] Bajhaiya AK, Moreira JZ, Pittman JK (2017). Transcriptional engineering of microalgae: prospects for high-value chemicals. Trends Biotechnol..

[3] Pulz O, Gross W (2004). Valuable products from biotechnology of microalgae. Appl. Microbiol. Biotechnol..

[4] Cordero BF, Obraztsova I, Couso I, Leon R, Vargas MA, Rodriguez H (2011). Enhancement of lutein production in *Chlorella sorokiniana* (Chorophyta) by improvement of culture conditions and random mutagenesis. Mar. Drugs.

[5] Kim M, Ahn J, Jeon H, Jin E (2017). Development of a *Dunaliella tertiolecta* strain with increased zeaxanthin content using random mutagenesis. Mar. Drugs.

[6] Saini DK, Chakdar H, Pabbi S, Shukla P (2019). Enhancing production of microalgal biopigments through metabolic and genetic engineering. Crit. Rev. Food Sci. Nutr..

[7] León-Bañares R, González-Ballester D, Galván A, Fernández E (2004). Transgenic microalgae as green cell-factories. Trends Biotechnol..

[8] Doron L, Segal Na, Shapira M (2016). Transgene expression in microalgae-from tools to applications. Front. Plant Sci..

[9] Davies JP, Weeks DP, Grossman AR (1992). Expression of the arylsulfatase gene from the β 2-tubulin promoter in *Chlamydomonas reinhardtii*. Nucleic Acids Res..

[10] Schroda M, Blöcker D, Beck CF (2000). The *HSP70A* promoter as a tool for the improved expression of transgenes in Chlamydomonas. Plant J..

[11] Goldschmidt-Clermont M, Rahire M (1986). Sequence, evolution and differential expression of the two genes encoding variant small subunits of ribulose bisphosphate carboxylase/oxygenase in *Chlamydomonas reinhardtii*. J. Mol. Biol..

[12] Kindle KL (1987). Expression of a gene for a light-harvesting chlorophyll a/b-binding protein in *Chlamydomonas reinhardtii*: effect of light and acetate. Plant Mol. Biol..

[13] Heitzer M, Zschoernig B (2007). Construction of modular tandem expression vectors for the green alga *Chlamydomonas reinhardtii* using the Cre/Iox-system. Biotechniques.

[14] Kimura M, Manabe K, Abe T, Yoshida S, Matsui M, Yamamoto YY (2003). Analysis of hydrogen peroxide-independent expression of the high-light-inducible ELIP2 gene with the aid of the ELIP2 promoter-luciferase fusion. Photochem. Photobiol..

[15] Park S, Lee Y, Lee J-H, Jin E (2013). Expression of the high light-inducible *Dunaliella* LIP promoter in *Chlamydomonas reinhardtii*. Planta.

[16] Qing G, Ma L-C, Khorchid A, Swapna G, Mal TK, Takayama MM (2004). Cold-shock induced high-yield protein production in *Escherichia coli*. Nat. Biotechnol..

[17] Kong F, Yamaoka Y, Ohama T, Lee Y, Li-Beisson Y (2019). Molecular genetic tools and emerging synthetic biology strategies to increase cellular oil content in *Chlamydomonas reinhardtii*. Plant Cell Physiol..

[18] Manuell AL, Beligni MV, Elder JH, Siefker DT, Tran M, Weber A (2007). Robust expression of a bioactive mammalian protein in *Chlamydomonas* chloroplast. Plant Biotechnol. J..

[19] Rasala BA, Muto M, Lee PA, Jager M, Cardoso RMF, Behnke CA (2010). Production of therapeutic proteins in algae, analysis of expression of seven human proteins in the chloroplast of *Chlamydomonas reinhardtii*. Plant Biotechnol. J..

[20] Baier T, Kros D, Feiner RC, Lauersen KJ, Müller KM, Kruse O (2018). Engineered fusion proteins for efficient protein secretion and purification of a human growth factor from the green microalga *Chlamydomonas reinhardtii*. ACS Synth. Biol..

[21] Davies PL, Baardsnes J, Kuiper MJ, Walker VK (2002). Structure and function of antifreeze proteins. Philos. Trans. R. Soc. Lond. B: Biol. Sci..

[22] Kim HJ, Lee JH, Hur YB, Lee CW, Park S-H, Koo B-W (2017). Marine antifreeze proteins: structure, function, and application to cryopreservation as a potential cryoprotectant. Mar. Drugs.

[23] Jung G, Lee C-G, Kang S-H, Jin E (2007). Annotation and expression profile analysis of cDNas from the Antarctic diatom *Chaetoceros neogracile*. J. Microbiol. Biotechnol..

[24] Gwak IG, sic Jung W, Kim HJ, Kang S-H, Jin E (2010). Antifreeze protein in Antarctic marine diatom, *Chaetoceros neogracile*. Mar. Biotechnol..

[25] Gwak Y, Jung W, Lee Y, Kim JS, Kim CG, Ju J-H (2014). An intracellular antifreeze protein from an Antarctic microalga that responds to various environmental stresses. FASEB J..

[26] Bayer-Giraldi M, Uhlig C, John U, Mock T, Valentin K (2010). Antifreeze proteins in polar sea ice diatoms: diversity and gene expression in the genus *Fragilariopsis*. Environ. Microbiol..

[27] Harris EH (2013). The Chlamydomonas sourcebook: a comprehensive guide to biology and laboratory use.

[28] Higo K, Ugawa Y, Iwamoto M, Korenaga T (1999). Plant cis-acting regulatory DNA elements (PLACE) database: 1999. Nucleic Acids Res..

[29] Chow C-N, Zheng H-Q, Wu N-Y, Chien C-H, Huang H-D, Lee T-Y (2016). PlantPAN 2.0: an update of plant promoter analysis navigator for reconstructing transcriptional regulatory networks in plants. Nucleic Acids Res..

[30] Lescot M, Déhais P, Thijs G, Marchal K, Moreau Y, Van de Peer Y (2002). PlantCARE, a database of plant cis-acting regulatory elements and a portal to tools for in silico analysis of promoter sequences. Nucleic Acids Res..

[31] Solovyev VV, Shahmuradov IA, Salamov AA (2010). Identification of promoter regions and regulatory sites. Methods Mol. Biol..

[32] Baek K, Lee Y, Nam O, Park S, Sim SJ, Jin E (2016). Introducing *Dunaliella* LIP promoter containing light‐inducible motifs improves transgenic expression in *Chlamydomonas reinhardtii*. Biotechnol. J..

[33] Berthold P, Schmitt R, Mages W (2002). An engineered *Streptomyces hygroscopicus* aph 7′′ gene mediates dominant resistance against hygromycin B in *Chlamydomonas reinhardtii*. Protist.

[34] Shao N, Bock R (2008). A codon-optimized luciferase from *Gaussia princeps* facilitates the *in vivo* monitoring of gene expression in the model alga *Chlamydomonas reinhardtii*. Curr. Genet..

[35] Valledor L, Furuhashi T, Hanak A-M, Weckwerth W (2013). Systemic cold stress adaptation of *Chlamydomonas reinhardtii*. Mol. Cell. Proteomics.

[36] Li L, Peng H, Tan S, Zhou J, Fang Z, Hu Z (2019). Effects of early cold stress on gene expression in *Chlamydomonas reinhardtii*. Genomics.

[37] Wurdinger T, Badr C, Pike L, De Kleine R, Weissleder R, Breakefield XO (2008). A secreted luciferase for *ex vivo* monitoring of *in vivo* processes. Nat. Methods.

[38] Kim J, Liu L, Hu Z, Jin E (2018). Identification and functional analysis of the *psaD* promoter of *Chlorella vulgaris* using heterologous model strains. Int. J. Mol. Sci..

[39] Baker SS, Wilhelm KS, Thomashow MF (1994). The 5′-region of *Arabidopsis thaliana cor15a* has cis-acting elements that confer cold-, drought-and ABA-regulated gene expression. Plant Mol. Biol..

[40] White TC, Simmonds D, Donaldson P, Singh J (1994). Regulation of BN115, a low-temperature-responsive gene from winter *Brassica napus*. Plant Physiol..

[41] Jiang C, Iu B, Singh J (1996). Requirement of a CCGAC cis-acting element for cold induction of the BN115 gene from winter *Brassica napus*. Plant Mol. Biol..

[42] Cheng L, Gao X, Li S, Shi M, Javeed H, Jing X (2010). Proteomic analysis of soybean [*Glycine max* (L.) Meer.] seeds during imbibition at chilling temperature. Mol. Breed.

[43] Lindlöf A, Bräutigam M, Chawade A, Olsson O, Olsson B (2009). *In silico* analysis of promoter regions from cold-induced genes in rice (*Oryza sativa* L.) and *Arabidopsis thaliana* reveals the importance of combinatorial control. Bioinformatics.

[44] Lauersen KJ, Kruse O, Mussgnug JH (2015). Targeted expression of nuclear transgenes in *Chlamydomonas reinhardtii* with a versatile, modular vector toolkit. Appl. Microbiol. Biotechnol..

[45] Lauersen KJ, Willamme R, Coosemans N, Joris M, Kruse O, Remacle C (2016). Peroxisomal microbodies are at the crossroads of acetate assimilation in the green microalga *Chlamydomonas reinhardtii*. Algal Res..

[46] Phillips JR, Dunn MA, Hughes MA (1997). mRNA stability and localisation of the low-temperature-responsive barley gene family blt14. Plant Mol. Biol..

