# Supplementary material for: Heterologous Gene Expression System Using the Cold-Inducible CnAFP Promoter in Chlamydomonas reinhardtii
Source: J Microbiol Biotechnol. 2020 Aug 15;30(11):1777–84. doi: 10.4014/jmb.2007.07024 (PMC9728157; doi:10.4014/jmb.2007.07024)

https://doi.org/10.4014/jmb.2007.07024

Table S1. PCR primers used in this study.

Purpose	Primer name (direction)	Sequence (5' to 3')
Sequence isolation & confirmation	pCnAFP_full_length (forward)	TCCGACTATACATCCCCCACACCCAACATG
	pCnAFP_full_length (reverse)	GTTTTGTGCTTTCTTGAAATTATGAGCTTT
	pCnAFP_inner_full_length (forward)	GTGCAATGCCCTGTGCTTGCTGTAGAGGCA
	pCnAFP_inner_full_length (reverse)	CTTTAGTTTTCCACACACCCAACATCCGCA
Vector construction	pCnAFP_full_length_SpeI (forward)	ACTAGT TCCGACTATACATCCCCCACACCCA
	pCnAFP_full_length_KpnI (reverse)	GGTACC GTTTTGTGCTTTCTTGAAATTATGAGCT
	pCnAFP_900bp_SpeI (forward)	ACTAGT TCTAGGCACCACGTCCCATGTTGA
	pCnAFP_600bp_SpeI (forward)	ACTAGT AATAATGTGACAGCACATATTTG
	pCnAFP_477bp_SpeI (forward)	ACTAGT TCGATACATGATTTAAATAACTTG
	pCnAFP_300bp_SpeI (forward)	ACTAGT AAATCAAACTGCAGAACTCTCTAAG
Transformant colony PCR	Vector_GLuc_colony_PCR (forward)	GCATTTAGTGCAATGCCCTGTGC
	Vector_GLuc_colony_PCR (reverse)	TGGCCCTGGATCTTGCTGGC
	Vector_mVenus_colony_PCR (forward)	GCGAACAATCACTAGCAGTGCAC
	Vector_mVenus_colony_PCR (reverse)	CCAATCGGGGTGTTCTGCT
	HygR_colony_PCR (forward)	ATGATTCCTACGCGAGCCTG
	HygR_colony_PCR (reverse)	ATCCGGCTCATCACCAGGTA
Quantitative real-time PCR	GLuc_qRT_PCR (forward)	GGCGAGGCCATCGTGGACAT
	GLuc_qRT_PCR (reverse)	TGGCCCTGGATCTTGCTGGC

	mVenus_qRT_PCR (forward)	ACGACGGCAACTACAAGACC
	mVenus_qRT_PCR (reverse)	CCAATCGGGGTGTTCTGCT
	RACK1_qRT_PCR (forward)	GGCTGGGACAAGATGGTCAA
	RACK1_qRT_PCR (reverse)	GAGAAGCACAGGCAGTGGAT

Table S2. Transcriptional regulatory elements in the *CnAFP* promoter

**Name**	**Function(s)**	**Position**
**LTRE**	low-temperature response expression	–1,224 (CCGAC)<br>–1,058 (GTCGG)
**MYCCONSENSUSAT**	dehydration and water stress response motifs<br>putative low-temperature responsive expression	–1,164 (CATATG)<br>–1,074 (CAAGTG)<br>–694 (CAGTTG)<br>–45 (CATTTG)
**ACGTERD1**	dehydration and water stress response motifs	–1,129, –848, –794 (ACGTT)
**MYB1AT**	dehydration and water stress response motifs	–561 (TAAACCACT)
**MYBCORE**	dehydration and water stress response motifs	–694 (CAGTTGAT)
**MYB2CONSENSUSAT**	dehydration and water stress response motifs	–1,193 (AACTGT)
**ACE**	*cis*-acting regulatory element involved in light responsiveness	–1,132 (ACTACGTTGA)
**G-box**	*cis*-acting regulatory element involved in light responsiveness	–891 (CACGTC)<br>–849 (CACGTTCA)<br>–795 (CACGTT)
**GT-1 motif**	*cis*-acting regulatory element involved in pathogen- and NaCl-induced expression	–1,028 (GGTAAT)
**CAAT-box**	common *cis*-acting regulatory element in promoter and enhancer regions	–1,032, –613, –429, –324, –159, –95, –84 (CAAT)<br>–602, –72 (CAAAT)<br>–548 (CCAATT)<br>–51 (CAATT)
**TATA-box**	core promoter element around –30 bp of transcription start	–1,219, –1,078, –640 (TATA)<br>–1,098, –570, –360 (TTTTA)<br>–374, –100 (TAATA)<br>–307 (CCTCTAAAAAT)<br>–224 (TTTTAAAAA)
**5'UTR Py-rich stretch**	*cis*-acting regulatory element conferring high transcription levels	–525 (TTTCGTCTCT)

Fig. S1. mRNA levels in *pCnAFP_GLuc* transformants according to the time pass after exposing at 10°C. (A) *GLuc*; (B) *RACK1* (reference gene). The relative mRNA level of *GLuc* calculated by *RACK1* was presented in **Figure 2C**.

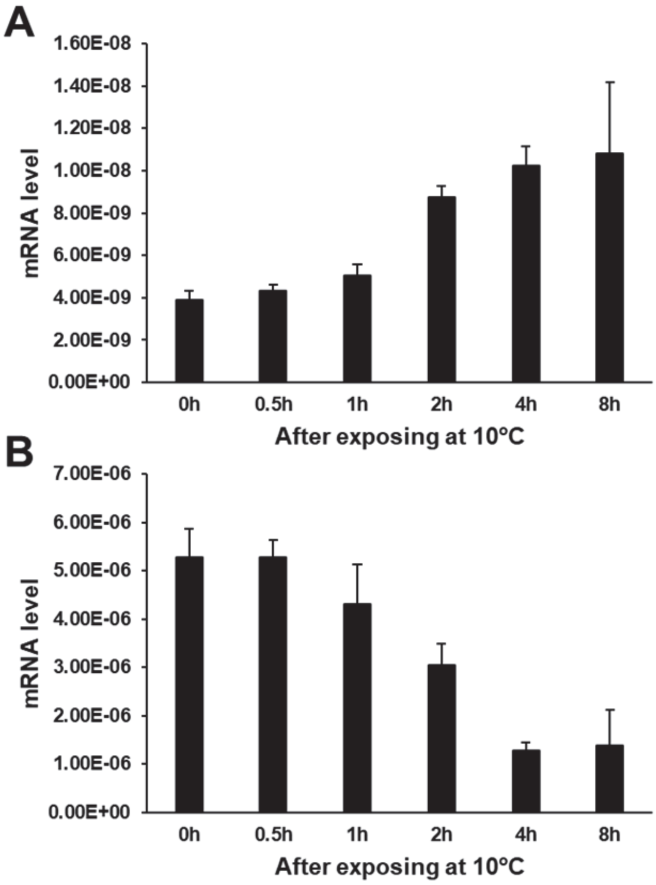

Fig. S2. Fluorescence images of mVenus transformants after low temperature (10°C) treatment (0h, 4h, and 8h). Merged images of mVenus (emission at 537–559 nm / excitation at 502–522 nm) and auto-fluorescence (emission at 603–648 nm / excitation at 563–588 nm) were shown. Yellow-circles indicate the cells presented in **Figure 5E**. Each scale bar is 10 μm.

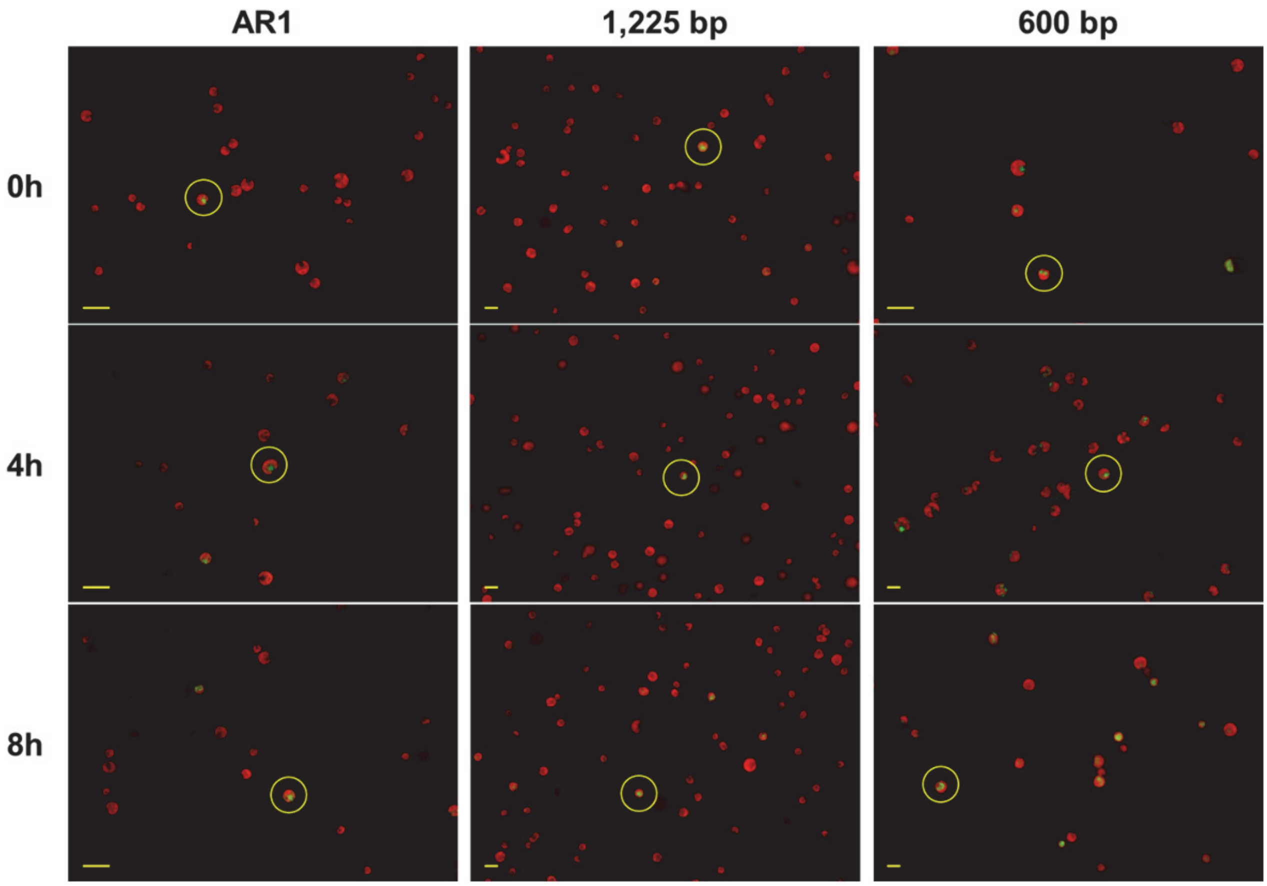

Supplement: Supplementary file 1 [file JMB-30-11-1777-supple.pdf]
